# Synthetic Lateral Inhibition in Periodic Pattern Forming Microbial Colonies

**DOI:** 10.1021/acssynbio.0c00318

**Published:** 2021-01-15

**Authors:** Salva Duran-Nebreda, Jordi Pla, Blai Vidiella, Jordi Piñero, Nuria Conde-Pueyo, Ricard Solé

**Affiliations:** † Institut de Biologia Evolutiva (CSIC-UPF), 08003 Barcelona, Spain; ‡ ICREA-Complex Systems Lab, Universitat Pompeu Fabra, 08003 Barcelona, Spain; § Evolution of Technology Lab, Institut de Biologia Evolutiva (CSIC-UPF), 08003 Barcelona, Spain; ∥ Santa Fe Institute, 1399 Hyde Park Road, Santa Fe, New Mexico 87501, United States

**Keywords:** synthetic biology, pattern formation, developmental biology, lateral inhibition

## Abstract

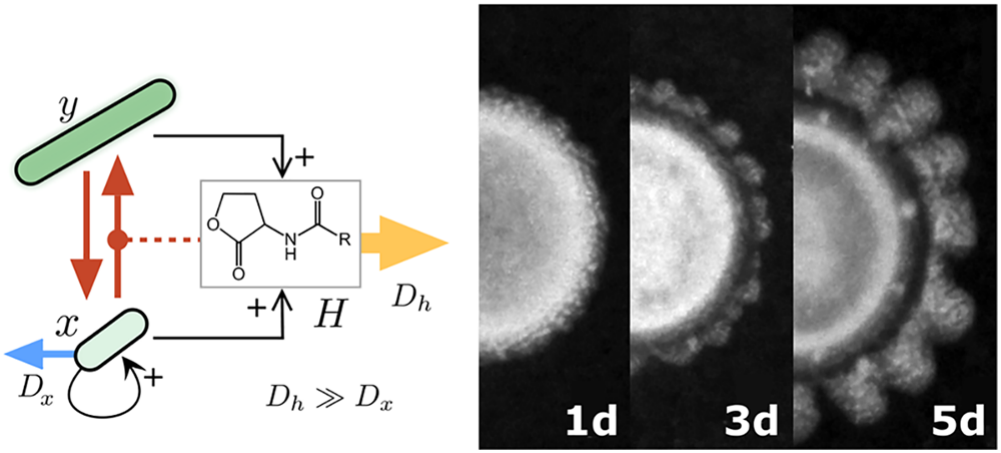

Multicellular entities are characterized by intricate spatial patterns, intimately related to the functions they perform. These patterns are often created from isotropic embryonic structures, without external information cues guiding the symmetry breaking process. Mature biological structures also display characteristic scales with repeating distributions of signals or chemical species across space. Many candidate patterning modules have been used to explain processes during development and typically include a set of interacting and diffusing chemicals or agents known as *morphogens*. Great effort has been put forward to better understand the conditions in which pattern-forming processes can occur in the biological domain. However, evidence and practical knowledge allowing us to engineer symmetry-breaking is still lacking. Here we follow a different approach by designing a synthetic gene circuit in *E. coli* that implements a local activation long-range inhibition mechanism. The synthetic gene network implements an artificial differentiation process that changes the physicochemical properties of the agents. Using both experimental results and modeling, we show that the proposed system is capable of symmetry-breaking leading to regular spatial patterns during colony growth. Studying how these patterns emerge is fundamental to further our understanding of the evolution of biocomplexity and the role played by self-organization. The artificial system studied here and the engineering perspective on embryogenic processes can help validate developmental theories and identify universal properties underpinning biological pattern formation, with special interest for the area of synthetic developmental biology.

The rise of multicellular life forms defines one of the major transitions in evolution, requiring novel ways of organization grounded in the cooperative interactions among single cells. The emergence of developmental processes and gene regulatory networks provided a flexible source of morphological diversity, facilitated by a number of physicochemical generative mechanisms.^1−3^ These mechanisms allow the emergence of long-range order out of locally interacting cells, and they often rely on signaling molecules diffusing in space,^2,4^ particularly at early stages of development.^1,5−8^

Embryogenesis often involves breaking different types of symmetry in a growing multicellular creature, such as left–right or ventral–dorsal distinctions in body plans.^9^ This requires a pattern formation mechanism able to create long-range order from an initially isotropic or homogeneous arrangement of cells, operating without exogenous chemical cues provided by the environment. A separate class of patterning mechanisms that have received great attention are those capable of generating regular structures at a characteristic length or scale, with repeating structures such as pigmentation spots^10^ or skeletal primordia^11^ arranged in a regular manner. A recurring element between different mechanisms of pattern formation is a short-range activation coupled with long-range inhibition.^12^ This core motif drives the local maintenance of an active region and the deactivation of the other domains at a characteristic distance. Lateral inhibition has been found to be naturally implemented though direct signaling to neighboring cells,^13^ local depletion of a resource or signal field,^14−16^ as well as competing sources of movement inducing signals.^17,18^

A very simple and elegant mechanism for the emergence of long-range order out of homogeneous systems also capable of producing regular structures was formulated by Alan Turing.^19,20^ Turing proposed that a system composed of two diffusing and interacting molecules (an activator and an inhibitor) could explain how an initially homogeneous state could lead to regular macroscopic structures by means of amplification of small perturbations. Specifically, Turing showed that under some mathematical conditions, reaction-diffusion systems can become unstable when incorporating strong differences in rates of diffusion between molecules.^19,21−24^

Since their inception, Turing patterns have come to be regarded as a paradigmatic example of simple systems yielding complex features and have been successfully found in both physical and chemical systems,^25−28^ and strong evidence has been found in biology. This includes skin pigmentation in animals,^29−35^ primordia of skeletal elements,^36−38^ palatal ridges,^39^ teeth formation,^40^ establishment of hair follicles,^41^ and ecological systems.^42−47^

Another possibility for inquiry into pattern formation is given by synthetic biology,^48−51^ which tries to artificially construct functions and features by splicing together gene sequences that do not naturally coexist.^52−54^ The synthetic biology approach has had crucial successes in developing some pattern formation mechanisms of symmetry breaking,^55^ gradient-based positional information developmental systems,^56,57^ and self-organized systems,^50,58^ although these have proven to be more elusive to engineer.^59−61^

Here we report a novel way of designing a synthetic lateral inhibition mechanism using bacteria that involves communication, cell elongation, adhesion, and growth inhibition. In order to implement signaling in our system, we make use of the widely studied quorum sensing genes from *V. fisherii*, which drive the expression of two proteins that change the physical properties of cells. More precisely, cell elongation and inhibition are provided by *MinC* expression, an endogenous *E. coli* protein which precludes septum formation, leading to longer cells that are metabolically active but have reduced biomass growth and cannot divide. Adhesion is introduced through a chimeric protein based on *JunA*,^62^ capable of homodimerizing on the outer side of cells with *JunA* of neighboring cells.

We propose that this system can be fundamentally simplified to three compartments (morphogens): a population of cells that is regular-sized, can divide and diffuse (*x*), a population of elongated and adhesive cells that is unable to divide or diffuse (*y*), and a rapidly diffusible quorum sensing lactone (*H*) that acts as the carrier of long-range inhibition. The lateral inhibition system presented here uses the physical embodiment of cells imposed by a synthetic differentiation process (*x* → *y*) to consistently break the symmetry during colony growth. Outward growths of cellular density are preceded by cohesive bundles of cells with similar orientation. This effect is present at the edge of the colony and impacts branching after the characteristic size has been surpassed. Additionally, we propose and explore an PDE model able to qualitatively reproduce some of the features of the system, including periodic pattern formation and symmetry breaking. This research provides an important milestone in the establishment of mutual feedbacks between experimental embryology, modeling of pattern formation, and synthetic strategies to reconstruct putative mechanisms and interactions.

## Materials and Methods

### DNA Constructs and Plasmids

Final genetic constructs used in this work were generated using the standard biobrick cloning techniques and enzymes: *Eco*RI/XbaI/SpeI/*Pst*I restrictases and T4 DNA ligase (New England Biolabs, USA). Some DNA sequences were provided by the iGEM 2010 spring collection, including *LuxR* (C0062), *LuxI* (C0161), *pLux* (R0062), *MinC* (K299806), *GFP* (E0040), constitutive promoter (J23100), bidirectional terminator (B0014) and RBSs (B0033, B0034). *JunA* was formatted to biobrick standard 10 from a coding sequence kindly provided by L.A. Fernández. The inefficient Lux promoter *pL40* was created *de novo* by primer hybridization (Sigma-Aldrich, USA). See Supporting Information for sequences of all used DNA pieces.

Genetic devices were split between two plasmids pSB1AC3 and pSB3K5, also obtained from the iGEM 2010 distribution, with high and high-intermediate copy numbers, respectively. The two plasmids harbor different origins of replication, and can coexist inside a single cell. All final constructs were sequenced by the PRBB core facilities.

### Bacterial Strains and Growth Conditions

Cloning procedures were carried out in *E. coli* Top10 strain (Invitrogen, USA). Final essays were performed in *E. coli* UT5600 kindly provided by L.A. Fernández.

Colony essays were performed as follows: UT5600 cells harboring each device were fresh plated overnight from a glycerinate stored at −80 °C, a single colony was then grown in Lisogenic Broth (Sigma-Aldrich, USA) supplemented with Chloramphenicol and Kanamycin (Sigma-Aldrich, USA) for 5 h and diluted to Abs_660_ = 0.2. A small volume (2 μL) of the density adjusted cultures was dropped in the center of 5.5 cm Petri dishes, filled with 5.5 mL of LB Eiken agar (Eiken Chemical, Japan) at 0.4% w/v again supplemented with Chloramphenicol and Kanamycin and, when necessary, 10^–8^ M *N*-[β-ketocaproyl]-l-homoserine lactone (Cayman Chemical Company, USA). Inoculated plates were dried for 5 min and grown 14 h at 37 °C, then stored at 22 °C for 7 days, were data capture took place.

### Data Capture and Processing

Assessment of lactone concentration impact on strain growth was carried out in Synergy MX microplate reader (BioTek Instruments, USA), similarly to our previously described protocol.^63^ Photographs of colony pattern were taken daily with a Canon EOS with diffuse illumination. Initial and final state of the pattern formation process were captured by bright field and fluorescence microscopy with a Leica DMI6000B (Leica Mycrosystems, USA). Regularities in colony boundaries were characterized with Matlab 2013b polar transformation and FFT algorithms (MathWorks, USA). All images were processed with a background subtraction and brightness adjustment.

### Computational Model

Custom scripts were developed to simulate the set of differential equations described in this work. These were implemented with an Euler numerical integration and a time step of 0.01 time units. The spatial lattices are either 1D or hexagonal 2D, and were created using *numpy* and *networkx* libraries. Signal analysis (FFT) and visualization were carried out using the *scipy* and *Matplotlib.pyplot* libraries, respectively. All simulations were carried out until stability was reached (20 000 algorithm iterations). For the initial cell seeding in the *in silico* experiments, the central coordinates (3 lattice sites for 1D simulations or all lattice sites within distance 10 of the center of the lattice for 2D) were initialized with active cells *x* = 0.05 + a random uniform distribution between 0 and 0.05.

## Results

### Synthetic Pattern-Generator Design

**Figure 1:**
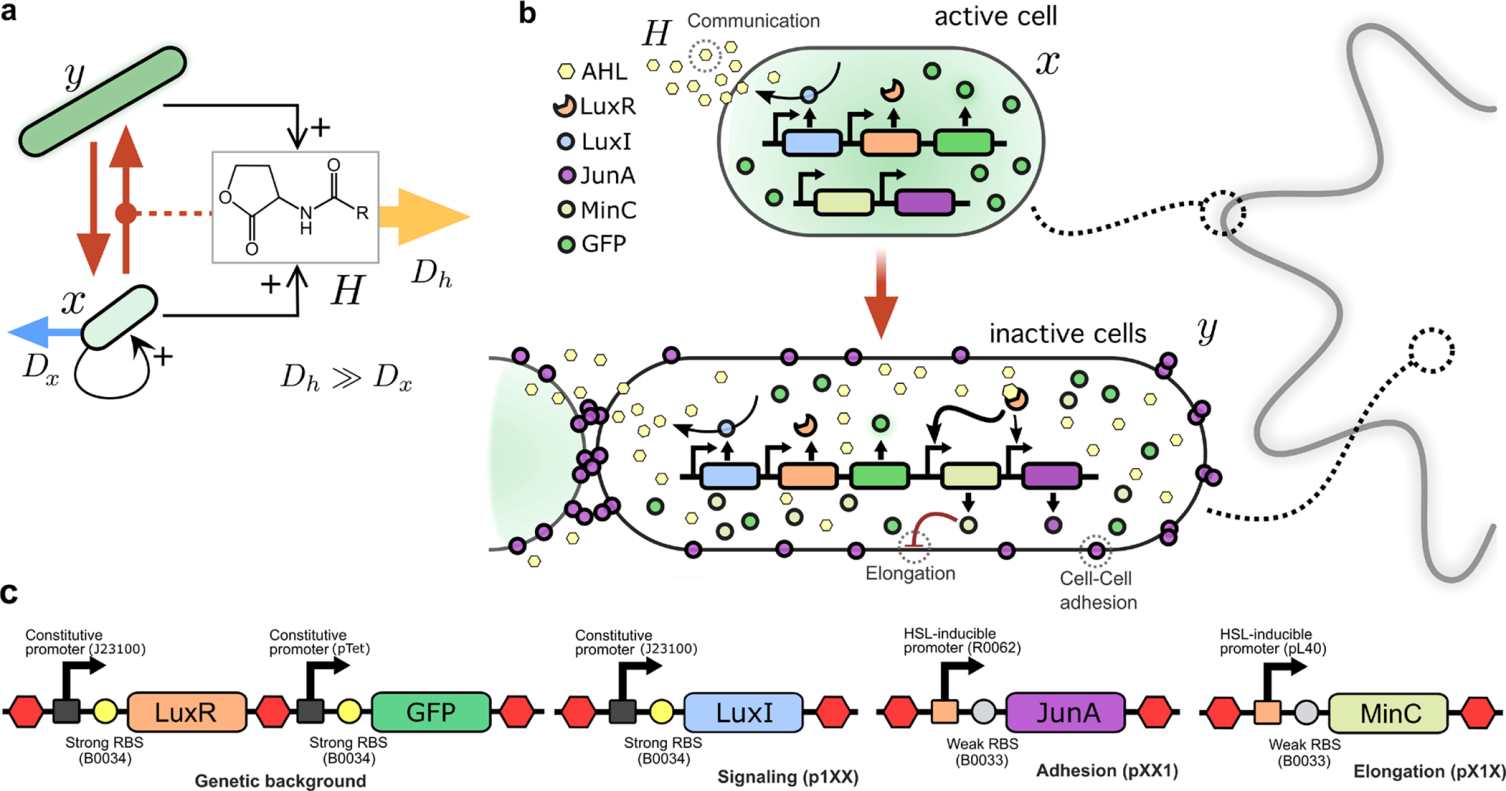
Engineering a symmetry breaking genetic device. (a) Interactions between the different elements of our engineered system. Here the initial cell population (*x*) can differentiate, under the presence of *H*, in an elongated phenotype that does not replicate (but also produces *H*) nor diffuse. The *H* molecule is an inhibitor acting both directly on *x* and indirectly through its effects on differentiation. (b) Core process in the lateral inhibition: as *x* cells locally sense more *H* they become more elongated and grow slower, eventually becoming unable to form the septum. (c) Genetic devices used in this work. A *GFP* reporter and Lux system receiver gene (*LuxR*) were present in all tested strains. The other three genes (*LuxI*, *MinC*, and *JunA*) were introduced in all possible combinations. Notation goes as follows, each bit indicates the presence or absence of a particular feature, from left to right: signaling, elongation, and adhesion.

The logic and components of our designed circuit are summarized in Figure 1. In Figure 1a, we display the interaction between the different species. Namely, our system is composed of three elements: a rapidly diffusing quorum sensing molecule (*H*), a population of *E. coli* capable of division and diffusion on the surface of an agar plate (*x*), and a phenotypic variant with increased length and enhanced adhesion that does not divide and has limited diffusion due to its size and the tendency to attach to other cells (*y*). These two *E. coli* phenotypes are distinct in their physical embodiment as well as their dynamical properties. *x* is the wildtype-sized population and grows at an exponential rate. Conversely, *y* represents a nongrowing population of elongated cells that can phenotypically regress to a standard-size phenotype at a very small rate. Both of these phenotypes constitutively synthesize the rapidly diffusing inhibitor molecule *H*.

**Figure 2:**
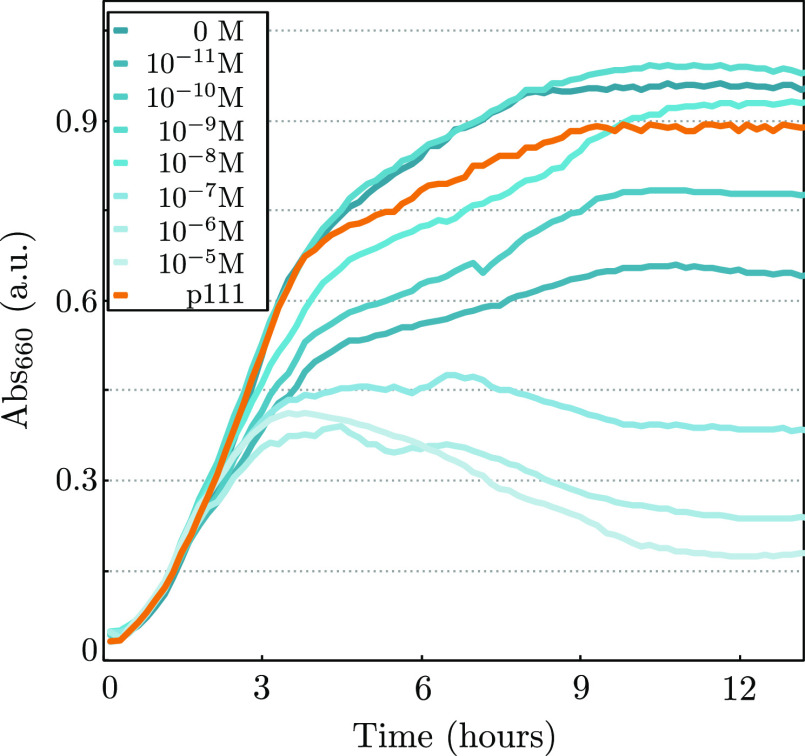
Bacterial growth and carrying capacity is influenced by lactone concentration. Characterization of the effect of *MinC* and *JunA* expression on the growth of synthetic cells. For different concentrations of the quorum sensing molecule, the construct harboring constitutive *LuxR* and conditional expression of *MinC* and *JunA*, shows different growth rates and carrying capacities (shades of blue, average of two independent replicates). As a control, a strain capable of endogenously synthesizing lactone, displays a density profile similar to 10^–9^ M of the previous case (orange).

In terms of genetic components, Figure 1c displays the five synthetic genes used in this study. First, as means of implementing communication, we make use of two genes the quorum sensing system of *V. fisherii*, widely used by the synthetic biology community.^56,57,59,64,65^ This is typically composed of a receptor protein (*LuxR*), able to enhance expression in specific promoter sequences in the presence of the ligand homoserine lactone (*H*), and the *LuxI* gene, able to synthesize the cognate molecule (*H*) from preexisting substrates. This family of ligand molecules can passively diffuse across the cellular membranes, reaching high concentrations naturally when cellular density surpasses a threshold. In our engineered system, these genes’ expression are driven by synthetic constitutive promoters from the biobricks collection (BBa_J23100), meaning that all cells shown are able to sense the presence of *H* and in the constructs with signaling, *H* synthesis is constant and its local concentration should be proportional to cellular density. We also introduce a cell–cell adhesion effect by the expression of a chimeric protein composed of the animal *JunA* coupled with an autotranslocator domain.^62^ This chimeric sequence is able to target the *E. coli* outer membrane, translocate to the extracellular side and homodimerize with *JunA* proteins expressed by other cells, increasing the sedimentation rates in liquid cultures of bacteria.^62^ This protein expression is controlled by a *pLux* promoter, a synthetic biobrick derivative (BBa_R0062) that positively enhances transcription in the presence of both *LuxR* and *H*. Diminished division and enhanced length are obtained by the expression of *MinC*, a natural protein involved in *E. coli* segmentation.^66^ In particular, increased levels of *MinC* have been shown to inhibit septum formation,^67^ which is crucial for cell division and has the secondary effect of creating elongated cells. Cells with increased *MinC* still grow in size albeit a slower rate^68^ (see Figure 2), leading to an elongated phenotype that can attain 2 orders of magnitude the typical cell length. Exogenous *MinC* expression is controlled in our system by a synthetic weak *pLux* promoter called *pL*40. This promoter displays positive control through *H* concentration but a lower leakiness and saturated expression than *pLux*. Finally, as a constitutive reporter in our system, we include a *pTetR* driven *GFP* gene, provided as a genetic background with *LuxR* and used as in confocal microscopy (see Materials and Methods). The Supporting Information include a detailed description of the DNA components and plasmids used in this study.

Figure 1b shows the core process driving pattern formation in our system, a phenotypical differentiation process (*x* → *y*) mediated through a diffusible inhibitor morphogen *H*. Increased levels of *H* ligand are internally interpreted by *LuxR*, which in turn drives the expression of *MinC* and *JunA*. As cells accumulate these two proteins they are less capable of forming septums, eventually becoming unable to divide, and elongate to a hundreds of times the mean wildtype length. At the same time, *JunA* expression coupled with increased surface per cell, synergistically boosts cell–cell adhesion. These two effects (increased mass and increased adhesion) severely reduce the diffusion of the differentiated cells *y*.

In order to test the effects of lactone-induced expression of *MinC* and *JunA* in the inhibition of growth, we monitored bacterial density at Abs_660_ in liquid cultures of different constructs, supplemented with increasing amounts of externally introduced lactone. Figure 2 shows how a strain able to express *MinC* and *JunA* in the presence of inducer but unable to constitutively synthesize the signaling molecule, displays varying rates of growth and carrying capacities dependent on the concentration of the signal. This necessarily implies that both a decrease in growth speed and a cell death response are mediated by *MinC* and *JunA*, suggesting that this mechanism can be responsible for an active lateral inhibition.

We propose that the operation of this patterning module adheres to the following sequence: as cells grow, they locally increase the concentration of the quorum sensing molecule, as *y* cells are not metabolically inert.^68^ As *H* rises, *x* cells become elongated and adhesive (*y* cells), which do not diffuse but still contribute to the local increase of *H*. Since *H* is a small molecule, it diffuses much more rapidly than *x*. Thus, the *H* front reaches further than the cell front and growth stalls. However, small perturbations at the edge of the cell front (protrusions of *x* and *y* cells that randomly break the symmetry of the colony) are amplified and grow faster than their neighboring regions. As this random perturbations grow in size, they inhibit other neighboring regions, creating a canonical local activation lateral inhibition mechanism of pattern formation.

### Nonhomogeneous Spatial Distributions of Cells Arise at the Intersection between Adhesion, Cell Signaling, and Filamentous Growth

**Figure 3:**
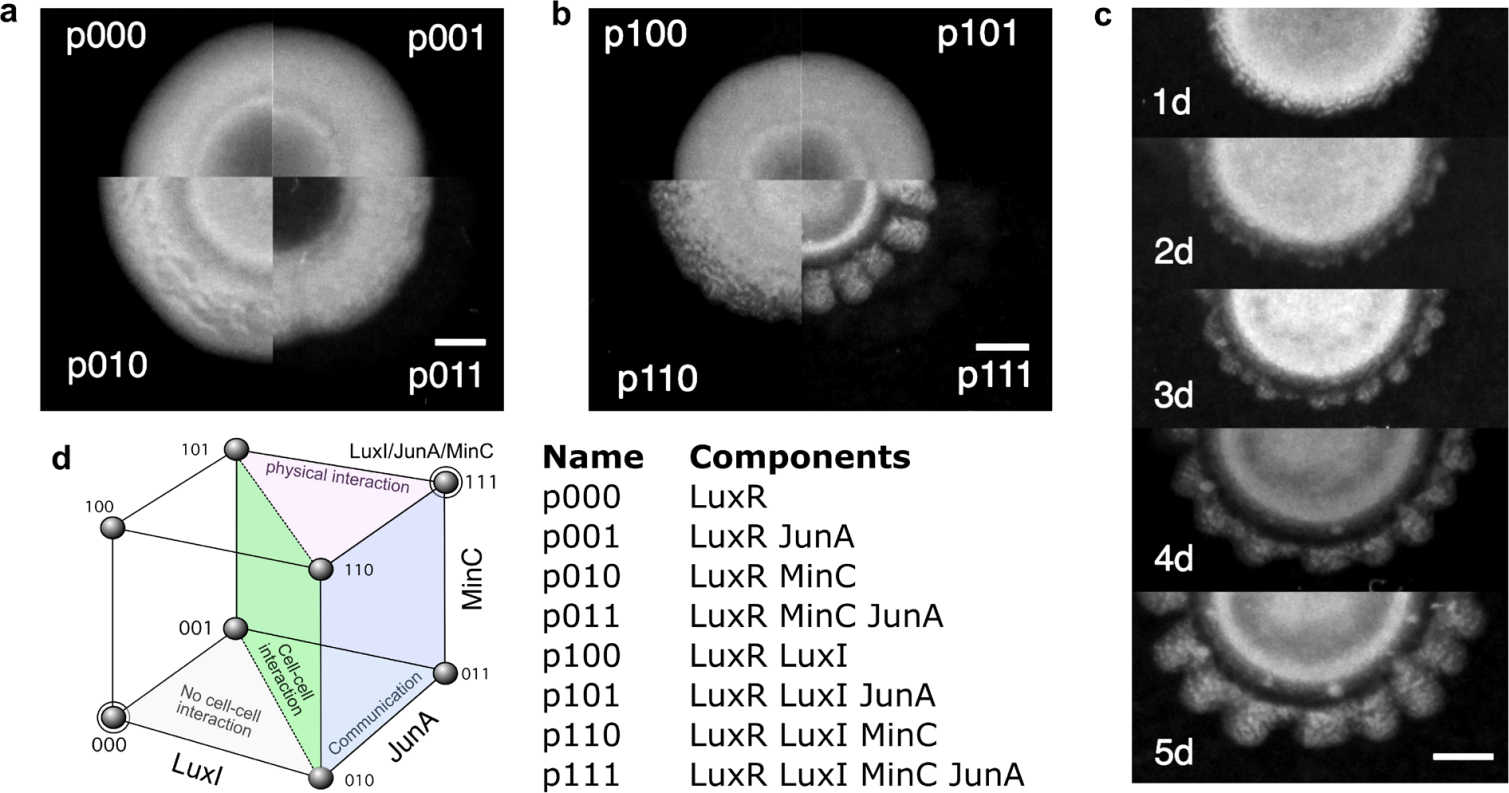
Regular patterns and symmetry breaking is only achieved at the intersection between communication, adhesion, and elongation. (a,b) Example colonies of all possible gene combinations: (a) shows all the constructs without the capability of synthesizing *H* (which was exogenously supplemented to the agar plates, see Materials and Methods), (b) shows all the constructs with signaling capabilities, the bottom rightmost quarter displays a colony with all genes (p111). (c) Daily progression time series of an example colony growth with communication, elongation and adhesion. (d) Summary table of the strain name and genetic components as well as a morphospace resulting from the combination of the three possible elements: the separation between individual behavior to cell–cell interactions (green surface), physical interaction (pink), and community communication/synchronization (blue). Scale bar in all pictures is 2 mm.

In this study we have assessed the impact of five genes (Figure 1c) in the colony growth of *E. coli* UT5600 strain, which typically develops into uniform circular colonies as time progresses (Figure 3a, p000). Expression of these genes engineers a differentiation process affecting cell morphology, growth rates, cell–cell adhesion and cell–cell signaling through quorum sensing. In order to explore the landscape of pattern formation capabilities of these genes, all possible combinations of them were constructed (Figure 3d), using the binary coordinates of the space (*LuxI*, *JunA*, *MinC*), where 1 indicates presence of the construct and 0 its absence, respectively.

An example colony for each of the eight gene combinations after 5 days of growth is shown in Figure 3a,b. Those conditions lacking the ability to synthesize the signaling molecule (p0XX) were externally supplemented with *H* in the Petri dish to saturation of *MinC* and *JunA* expression, but otherwise lack the spatial information given by the quorum sensing mechanism. Only when all three capabilities were included in the synthetic cells (*i.e.*, at the (1, 1, 1) vertex of our binary 3D space) spatial nonhomogenous distributions of cell densities were created, in stark contrast to the other conditions were isotropic growth took place. Figure 3c shows p111 colony growth every 24 h, from a uniform seeding of cells in an agar plate small perturbations in the colony front are observed at the second day of growth, which are continuously amplified in the subsequent days.

### Nonhomogenous Patterns Are Characterized by a Dominant Wavelength

**Figure 4:**
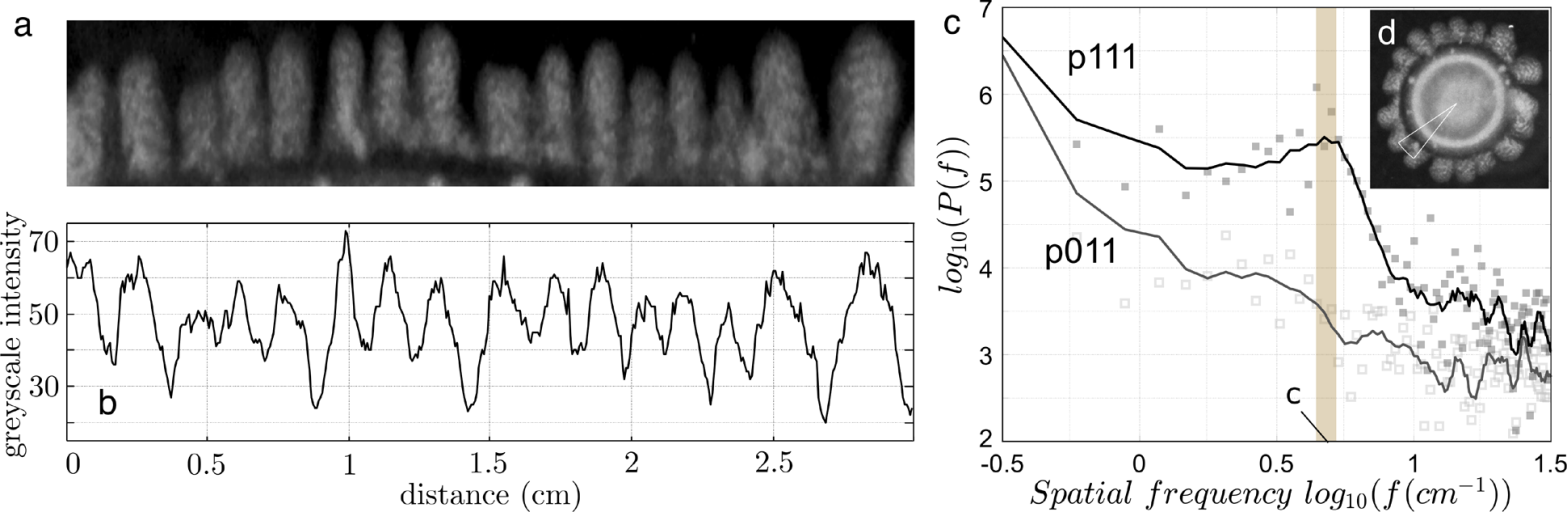
Characterization of the regular structures created by bacterial density. (a) Five-day colony profile after being transformed into polar coordinates. The intensity (a.u.) from the previous data is used as a surrogate of bacterial density close to the edge of the colony. A one-dimensional spatial set (b) is used to calculate the resulting power spectrum (c) showing the existence of a peak at a frequency value leading to a characteristic wavelength λ ∼ 0.2 cm (brown shading) and consistent with the scale observed in mini Petri-dishes (inset). Single colony assessment; for comparison we show the same analysis for a unstructured p011 colony (which lacks any characteristic scale).

As it can be seen in Figure 3b, the symmetry of the colony is broken in a regular fashion only when *LuxI*, *JunA*, and *MinC* are present (p111). Additionally, a characteristic scale of the pattern appears to be preserved along the colony growth process, as displayed in Figure 3e, where we show a time series. The presence of some characteristic length scale would point to a self-organizing pattern, implying some form of local activation coupled with long-range inhibition. In order to better characterize the higher order properties displayed by the growing structure, we analyzed the distribution of bacterial concentrations in a circumference centered at the estimated center of the colony. In order to do so, the bright field information on these colonies was transformed from Cartesian to polar coordinates (Figure 4a,b). A Fast Fourier Transform (FFT) algorithm was used to calculate the power spectrum *P*(*f*) of the spatial data using the brightfield gray scale intensity as a surrogate for bacterial density (Figure 4b). As expected, the one-dimensional mapping is affected by local fluctuations associated with deviations from a perfect circular shape, which tends to broaden the *P*(*f*). Nevertheless, a well-defined peak is observed corresponding to a characteristic wavelength of approximately λ ≈ 0.2 cm (Figure 4c) consistent with the observed length scale of the pattern displayed by the colony (inset Figure 4c). For comparison, the Power spectrum for a p011 colony is shown, which does not reveal any strong periodic signal.

### Branching Primordia and Long-Term Evolution

**Figure 5:**
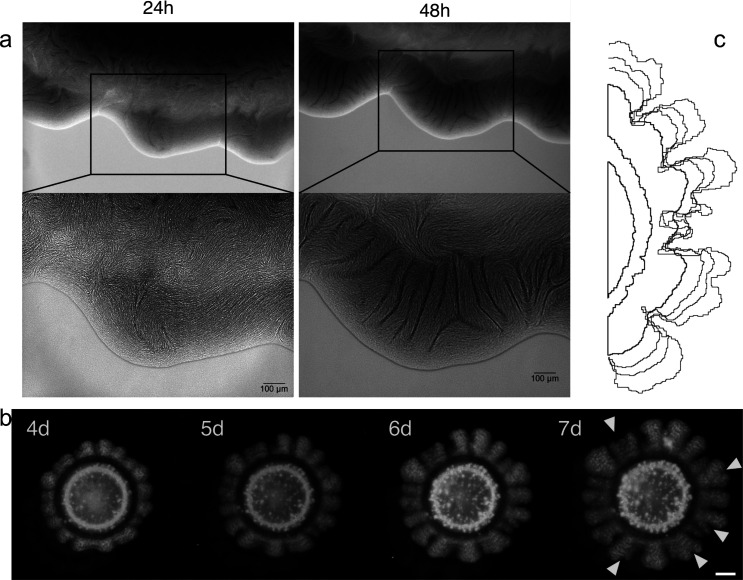
Small-scale and long-term pattern formation. (a) Displays the same region of a p111 strain colony after 24 and 48 h of growth in bright field microscopy. On the top, a wider region containing several branches, below the detail of cell bundles forming at the edge of the growing colony. (b) Time series of branch formation in older colonies. From the fourth day to the seventh we show the same p111 colony; once the zones of low density have been created they are not homogenized by the effects of diffusion. Marked with arrows are the new branching points: when domain width has surpassed a critical size, new troughs are created that branch the density. Scale bar represents 2 mm. (c) For this same experiment, overlapped edge colony detection with standard ImageJ libraries using a brightfield intensity threshold.

In order to assess the microscale properties of the pattern formation process we captured the first stages of colony growth with bright field and fluorescence microscopy (Figure 5a). We observed that when the whole set of genes is present, the starting symmetry imposed by the circular droplet of cells is broken as soon as 24 h with small perturbations to the circular shape. These primordia of the global pattern do not have the regularity displayed by the colony at later stages, and are characterized by the formation of cohesive bundles of cells with the same orientation (Figure 5a, bottom). Given that *E. coli* segmentation occurs perpendicular to the longest axis of the cell, the establishment of a collective orientation forcefully imposes growth in a preferred direction, which cells maintain in the following days. The synthetic cell adhesion features provided by *JunA* and *MinC* along with the local directionality imposed by the *E. coli* cell shapes can explain these features and might play some relevant role in the amplification mechanisms triggered by our synthetic symmetry-breaking device.

The long-term branching beyond the 5-day window used to determine characteristic scale reveals other qualitative properties that are consistent with a lateral inhibition process. In Figure 5b a series of snapshots are used to see how the regular branching is maintained, but also how in the long run the widest branches experience new bifurcations (Figure 5b, seventh day snapshot). At this point, new front instabilities might be taking place as a consequence of the lateral inhibition mechanism: since a characteristic λ_*c*_ is the only stable solution, structures with a larger wavelength will tend to split.^69^Figure 5c shows for the same colony in Figure 5b, the superimposed colony boundaries for different days, showing the long-term stability of the pattern: troughs of cell density are not blurred by the effects of diffusion, gliding, or cellular motility.

### Reaction-Diffusion Model

Putting together the evidence shown in the previous sections, we devised a reaction-diffusion mathematical model similar to those found in bacterial colony growth,^70−72^ where nutrient limitation can promote variations in colony morphology. The model contains the three main species described before: active cells (*x*), quorum sensing signal (*H*), and inactive elongated cells (*y*). Given our knowledge of the system, we propose that it can be formalized using the following set of ordinary differential equations:
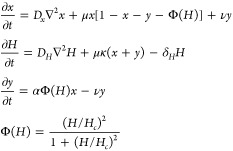
where only the first two species (active cells and homoserin lactone) can diffuse in space and thus have a standard diffusion operator. Inactive cells are drawn from the active population at a rate proportional to Φ(*H*), a standard Hill-function functional for genetic regulation (here we use the commonly assumed *n* = 2 as exponent). The *x* growth dynamics are essentially governed by a modified logistic growth (*μx*(1 – *x* – *y* – Φ(*H*))), and both cell types produce the signal at a linear rate (λ(*x* + *y*)), which decays exponentially at a rate proportional to δ_*H*_*H*. Finally, a small fraction of elongated can return to the active phenotype with a linear rate ν.

**Figure 6:**
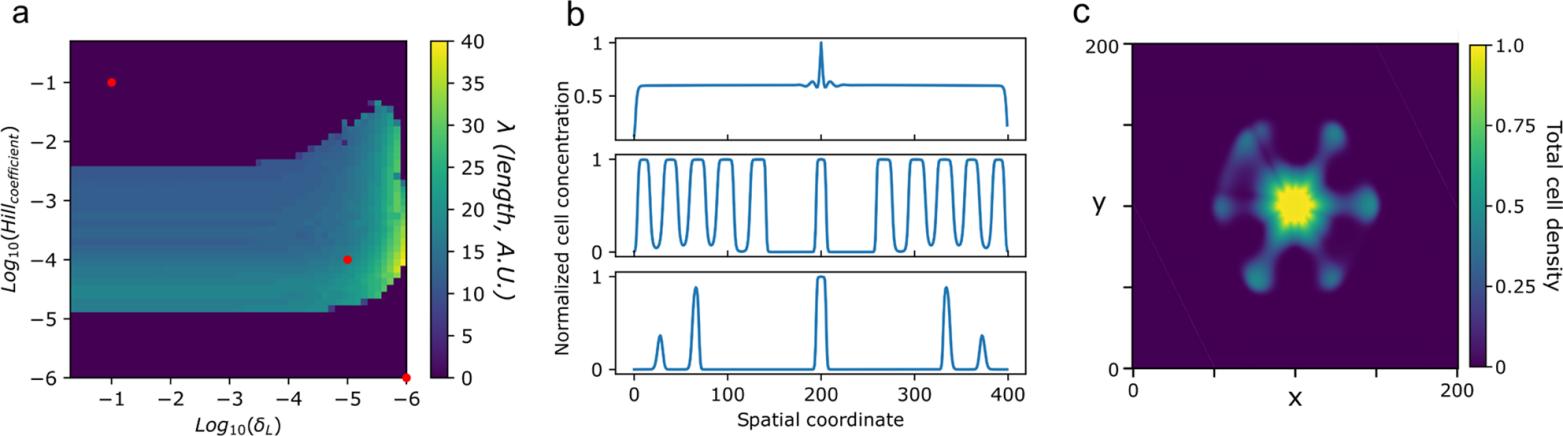
Pattern formation in our computational model of synthetic symmetry breaking in bacterial colonies. (a) Parameter sensitivity analysis of δ_*H*_ and *H*_*c*_ on the wavelength and stability of the formed structures (wavelength is here shown in units of normalized lattice site length). 51 × 51 parameter pairs, representing each 5 replicates of a 1D domain of 400 lattice elements and 2× 10^4^ algorithm iterations. Other parameters values are *D*_*x*_ = 0.005, *D*_*H*_ = 10^3^*D*_*x*_, α = 2, μ = 8, ν = 0.001, κ = 0.1. Wavelength was computed using *scipy* FFT and peak finding standard libraries. Phases were delimited using peak prominence in the power spectrum, using a prominence threshold value of 1.5. (b) In some regions of this parameter space, only traveling fronts are formed which cannot limit their own growth (top), while for other values the inhibition is stronger and damped oscillations are formed (bottom). Cell concentration profiles (*x* + *y*) over the 1D 400 lattice sites after random seeding in the center of the spatial domain and after 2 × 10^4^ algorithm iterations. (c) Symmetry breaking in a 2D 200 × 200 lattice implementation of the model (parameters used in this simulation are the same as mentioned previously except: μ = 2, *H*_*c*_ = 5, δ_*H*_ = 0.005).

In order to better understand the dynamical and stability features of our engineered system the set of ODEs was computationally implemented in custom *python* scripts and explored in terms of its parameters space, seeking to qualitatively reproduce the features observed in the experimental setup, namely a symmetry breaking mechanism that amplifies small perturbations on a propagating front and can create periodic arrangements of cellular densities. Figure 6a shows a systematic exploration of wavelength (λ) for two relevant parameters: the rate of homoserin lactone decay δ_*H*_ and the Hill constant that establishes lactone semiactivation concentration in the Hill-function (*H*_*c*_). These two parameters determine if any stable pattern is formed. In Figure 6b we show a traveling front (log_10_(δ_*H*_) = −1, log_10_(*H*_*c*_) = −1, top) a standing wave (log_10_(δ_*H*_) = −5, log_10_(*H*_*c*_) = −4, middle) and spatial damped oscillations (log_10_(δ_*H*_) = −6, log_10_(*H*_*c*_) = −6, bottom) marked as red dots in Figure 6a. Figure 6c shows an example pattern in 2D after seeding a central section of an hexagonal lattice with a small perturbation to a constant concentration (see Materials and Methods).

## Discussion

Turing’s initial proposal of a diffusion-driven pattern formation mechanisms was nothing short of revolutionary for the area of developmental biology. It kickstarted a decades long search for the interaction motifs and the molecular entities that might underlie such process. The Turing framework was later expanded and formalized by Gierer and Meinhardt, who described this mechanism in terms of local activation and long-range inhibition.^21^ This motif has since been expanded into networks of interacting morphogens,^73,74^ often including more than three compartments and making use of nondiffusible elements. This rich theoretical background found wide success in chemical systems,^25−28^ where chemical Turing patterns were reported and studied long before the biological domain. However, the molecular evidence for Turing-type mechanism in biological systems has been sparse up until very recently,^37−39^ casting doubts on the universality this mechanism.

Besides diffusion-driven instabilities, a wealth of pattern forming mechanisms have been proposed that can account for periodic distributions of morphogens: from gene regulatory networks in butterfly wings,^75^ Delta-Notch interaction in lateral inhibition,^13^ resource-sink models of reaction diffusion,^16^ coalescing migration,^18^ phase separation coupled with phenotypic transitions^76^ to morphogen diffusion informing spatial memory in skeletal limb primordial.^14^ While these studies are intrinsically valuable in exploring the landscape of possibilities, here we have taken another approach: constructing *de novo* a system capable of producing periodic structures using synthetic biology components. Synthetic biology offers a unique perspective into big standing questions in developmental biology and pattern formation. By attempting to manufacture ways to break isotropy we can better understand the complex molecular waltz sitting at the core of creating a new organism. New insights, mechanisms, and constraints might be encountered when attempting to replicate models of pattern formation, wether natural or those predicted by theory. This perspective has the added benefits of testing preconceived notions on how a system must operate.

In particular, a crucial element in the work presented here is the cellular embodiment and how cellular level properties might affect pattern formation at the larger scale.^77,78^ In our system, cells become elongated through the exogenous expression of MinC, driven by the concentration of a quorum sensing molecule homoserine lactone. The elongation has a synergistic effect with the surface presentation of the adhesion protein JunA, enhancing the capacity of cells to attach with one another and create cohesive bundles. Domains with cohesive arrangements of cells appear very soon during colony development, and their orientation directs the branching process at the edge of the colony. This level of detail in synthetic biology applications is often overlooked for the more simplistic and tractable computational perspective.^56,65^ However, an embodied perspective on synthetic pattern formation has plenty of theory to draw upon^1^ and can try to achieve symmetry breaking with the help of physical processes, not despite their existence. This, in turn, might drive the focus of synthetic biology from a computational perspective to a functional one,^53^ with toolkits developed to interface with known physical processes through the agent embodiment.

## Supporting Information Available

List of plasmids, plasmid feature maps, construct sequences and gene origin, characterization of *MinC* differentiation, long-term evolution of pattern forming colonies (PDF)
