# Supplementary material for: Synthetic Lateral Inhibition in Periodic Pattern Forming Microbial Colonies
Source: ACS Synth Biol. 2021 Jan 15;10(2):277–85. doi: 10.1021/acssynbio.0c00318 (PMC8486170; doi:10.1021/acssynbio.0c00318)
Supplement: Supplementary file 1 — sb0c00318_si_001.pdf [file sb0c00318_si_001.pdf]

Supplementary Material

Synthetic lateral inhibition in periodic pattern forming microbial colonies

Salva Duran-Nebreda*, Jordi Pla, Blai Vidiella, Jordi Piñero, Nuria Conde-Pueyo and Ricard Solé*

I. PLASMIDS, SEQUENCES AND PRIMERS

Name	Description	Ref.
pSB1AC3	Basic *biobrick* cloning vector. Contains Ampicillin and Chloramphenicol resistances and a pMB1 replication origin.	[1]
pSB3K5	Basic *biobrick* cloning vector. Contains a Kanamicin resistance marker and a p15A origin of replication.	[1]
pSB_pLuxJunA	Chimeric JunA expression regulated by a canonical Lux (R0062) promoter. Built onto a pSB1AC3 backbone.	[2, 3]
pSB_pLuxMinC	MinC expression regulated by a low efficiency RBS (B0033) and a low efficiency Lux promoter. Built onto a pSB1AC3 backbone.	
pSB_pLuxJunA_MinC	Concatenation of the two previous constructs. Built onto a pSB1AC3 backbone.	[2, 3]
pSBLuxRGFP	Plasmid with constitutive expression of LuxR and GFP. Built onto a pSB3K5 backbone.	
pSBLuxRLuxIGFP	Plasmid with constitutive expression of LuxR, LuxI and GFP. Built onto a pSB3K5 backbone.	

TABLE I: List of plasmids used in this article.

A. Plasmid Maps

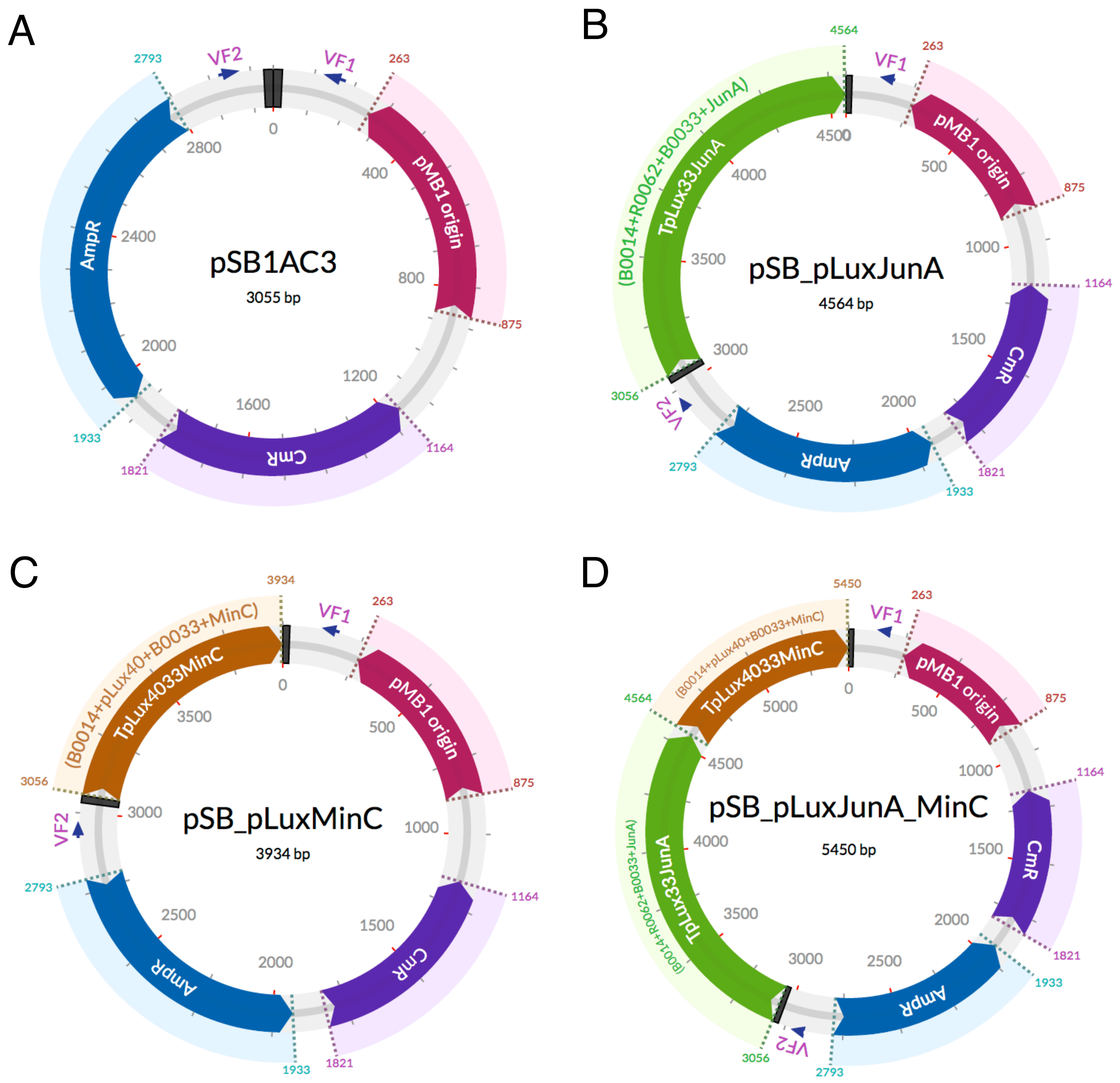

FIG. 1: **Plasmid feature maps for the pSB1AC3 family**. (A) Shows the genetic elements contained in an empty pSB1AC3 *biobrick* backbone. Grey regions correspond to the standard *biobrick* cloning sites (prefix and suffix). *VF1* and *VF2* correspond to the standard sequencing primers (reverse and forward respectively). In (B-D) we show the modified versions with our constructs.

B. Construct sequences

>Terminator (BBa_B0014) + pLux promoter (BBa_R0062) + weak RBS (BBa_B0033) + JunA autotransporter
TCACACTGGCTCACCTTCGGGTGGGCCTTTCTGCGTTTATATACTAGAGAGAGAATATAAAAAGCCA
GATTATTAATCCGGCTTTTTTATTATTTACTAGAGACCTGTAGGATCGTACAGGTTTACGCAAGAAA
ATGGTTTGTTATAGTCGAATAAATACTAGAGTCACACAGGACTACTAGATGAAATACCTATTGCCTAC
GGCAGCCGCTGGATTGTTATTACTCGCGGCCCAGCCGGCCATGGCGGACCGGATCGCCCGGCTCGAGG
AAAAAGTGAAAACCTTGAAAGCGCAAAACTCCGAGCTGGCGTCCACGGCCAACATGCTCAGGGAACAG
GTGGCACAGCTTAAACAGAAAGTCATGAACCACGAGGCCTCGGGGGCCGAATTTGTCGACGGTGCGCC
GGTGCCGTATCCGGATCCGCTGGAACCGATCGACAATTCAGCCGCAATTAGTATGGCAAATCCACGTC
CACCAACACCGCGGACACCTACGCCGGGTCCGGATCTGAATGTGGATAATGACCTGCGACCGGAGGCG
GGTAGCTACATTGCGAACCTTGCAGCAGCGAATACCATGTTCACCACGCGTCTGCATGAGCGTCTGGG
TAATACGTACTATACCGACATGGTGACGGGTGAGCAGAAACAAACCACTATGTGGATGCGCCATGAAG
GTGGTCATAATAAATGGCGTGATGGCAGCGGCCAGCTGAAAACCCAAAGCAATCGCTATGTTCTGCAA
CTGGGAGGCGATGTCGCGCAGTGGAGCCAAAACGGCAGCGACCGCTGGCATGTTGGGGTCATGGCGG
GATATGGCAACAGCGACAGCAAAACCATTTCCTCGCGAACCGGTTATCGTGCAAAAGCGAGTGTGAAC
GGATATAGCACAGGCCTCTATGCCACCTGGTATGCCGATGACGAGTCGCGTAATGGCGCGTATCTCGA
CAGTTGGGCGCAGTACAGCTGGTTTGATAACACAGTGAAAGGGGATGACTTACAAAGTGAATCCTATA
AATCAAAAGGATTTACCGCTTCACTGGAAGCTGGATACAAACACAAATTAGCTGAATTTAATGGCAGC
CAGGGAACGCGTAATGAATGGTATGTTCAGCCGCAAGCACAGGTTACCTGGATGGGAGTCAAAGCCG
ATAAGCACCGCGAAAGCAACGGAACCCTCGTTCATAGCAACGGTGATGGCAATGTTCAAACCCGACTT
GGCGTAAAAACCTGGCTGAAGAGCCACCATAAAATGGATGACGGTAAATCCCGCGAGTTCCAGCCGTT
TGTAGAAGTGAACTGGCTACATAACAGTAAGGATTTCAGCACCAGTATGGATGGCGTGTCTGTCACTC
AGGATGGAGCCCGAAATATTGCTGAGATAAAAACCGGGGTGGAAGGACAGCTAAATGCCAACCTGAA
TGTCTGGGGGAATGTGGGCGTTCAGGTTGCCGATAGGGGATATAATGACACCTCTGCAATGGTTGGC
ATTAAGTGGCAATTCTGA

>Terminator (BBa_B0014) + weak pLux promoter + weak RBS (BBa_B0033) + MinC (BBa_K299806)
TCACACTGGCTCACCTTCGGGTGGGCCTTTCTGCGTTTATATACTAGAGAGAGAATATAAAAAGCCA
GATTATTAATCCGGCTTTTTTATTATTTACTAGAGACCTGTAGGATCGTACAGGTTTACGCAAGAAA
ATGGTTTGTTATAGTCGAATAAATACTAGAGTCACACAGGACTACTAGATGTCAAACACGCCAATCGA
GCTTAAAGGCAGTAGCTTCACTTTATCTGTGGTTCATCTGCATGAGGCAGAACCTAAGGTTATCCATC
AGGCGCTGGAAGACAAAATCGCTCAGGCCCCCGCATTTTTAAAACATGCCCCCGTTGTACTCAACGTC
AGTGCACTGGAAGACCCGGTAAACTGGTCAGCGATGCATAAGGCGGTTTCGGCAACCGGTTTGCGGG
TTATTGGCGTAAGTGGCTGCAAAGATGCGCAACTTAAAGCCGAAATTGAAAAGATGGGGCTGCCTATC
CTGACGGAAGGAAAGGAAAAAGCGCCACGTCCAGCTCCCACACCGCAGGCTCCAGCGCAAAATACAAC
GCCGGTCACAAAAACGCGTTTAATAGATACCCCGGTGCGTTCCGGTCAGCGTATTTATGCTCCACAAT
GTGATCTGATTGTTACAAGCCACGTTAGCGCTGGGGCCGAATTGATTGCCGATGGGAACATTCATGTC
TATGGCATGATGCGCGGTCGTGCGCTGGCAGGGGCAAGTGGTGACCGGGAAACGCAAATATTTTGTA
CGAACCTGATGGCGGAACTGGTGTCCATCGCAGGTGAATACTGGCTGAGTGATCAAATCCCAGCAGAA
TTTTATGGCAAAGCGGCGCGACTGCAGTTAGTCGAAAACGCTTTGACCGTTCAACCGTTAAATTGA

>Terminator (BBa_B0014) + constitutive promoter (BBa_J23100) + RBS (BBa_B0034) + LuxR (BBa_C0062) + Terminator (BBa_B0014) + constitutive promoter (BBa_J23100) + RBS (BBa_B0034) + LuxI (BBa_C0161) + Terminator (BBa_B0014) + constitutive promoter (BBa_R0040) + RBS (BBa_B0034) + GFP (BBa_E0040)
TCACACTGGCTCACCTTCGGGTGGGCCTTTCTGCGTTTATATACTAGAGAGAGAATATAAAAAGCCA
GATTATTAATCCGGCTTTTTTATTATTTACTAGAGTTGACGGCTAGCTCAGTCCTAGGTACAGTGCT
AGCTACTAGAGAAAGAGGAGAAATACTAGATGAAAAACATAAATGCCGACGACACATACAGAATAATT
AATAAAATTAAAGCTTGTAGAAGCAATAATGATATTAATCAATGCTTATCTGATATGACTAAAATGGT
ACATTGTGAATATTATTTACTCGCGATCATTTATCCTCATTCTATGGTTAAATCTGATATTTCAATCCT
AGATAATTACCCTAAAAAATGGAGGCAATATTATGATGACGCTAATTTAATAAAATATGATCCTATAG
TAGATTATTCTAACTCCAATCATTCACCAATTAATTGGAATATATTTGAAAACAATGCTGTAAATAAA
AAATCTCCAAATGTAATTAAAGAAGCGAAAACATCAGGTCTTATCACTGGGTTTAGTTTCCCTATTCA
TACGGCTAACAATGGCTTCGGAATGCTTAGTTTTGCACATTCAGAAAAAGACAACTATATAGATAGTT
TATTTTTACATGCGTGTATGAACATACCATTAATTGTTCCTTCTCTAGTTGATAATTATCGAAAAATAA
ATATAGCAAATAATAAATCAAACAACGATTTAACCAAAAGAGAAAAAGAATGTTTAGCGTGGGCATGC
GAAGGAAAAAGCTCTTGGGATATTTCAAAAATATTAGGTTGCAGTGAGCGTACTGTCACTTTCCATTT
AACCAATGCGCAAATGAAACTCAATACAACAAACCGCTGCCAAAGTATTTCTAAGCAATTTTAACAG

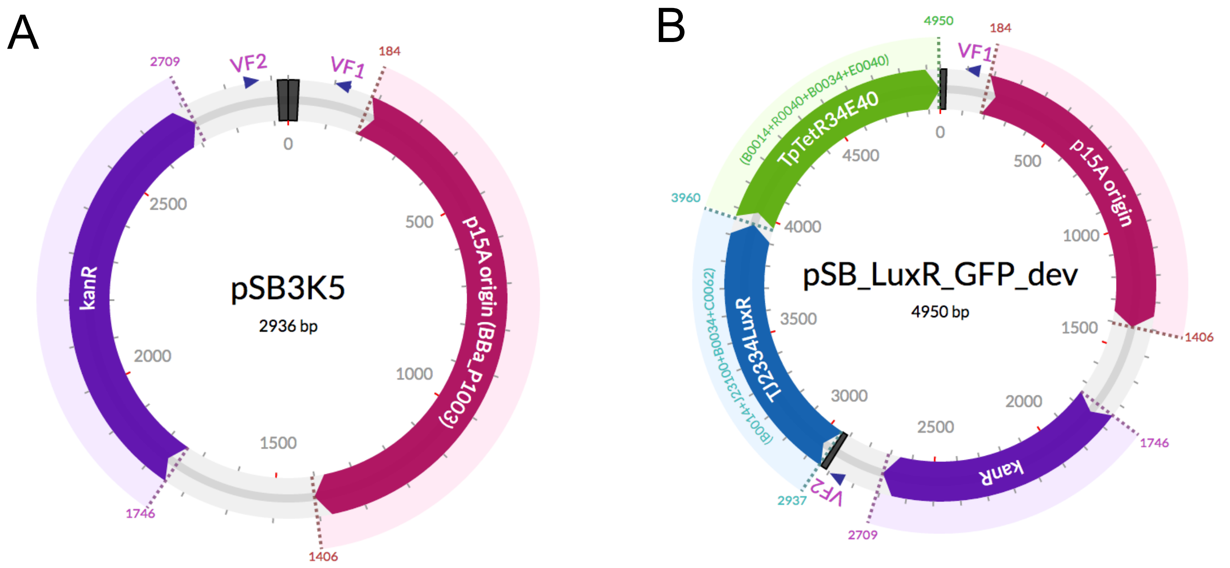

FIG. 2: **Plasmid feature maps for the pSB3K5 family**. (A) Shows the genetic elements contained in an empty pSB3K5 *biobrick* backbone. Grey regions correspond to the standard *biobrick* cloning sites (prefix and suffix). *VF1* and *VF2* correspond to the standard sequencing primers (reverse and forward respectively). In (B) we show the modified version with our constructs.

GAGCAATTGATTGCCCATACTTTAAAAATTAATAACACTGATAGTGCTAGTGTAGATCACTACTAGAG
TCACACTGGCTCACCTTCGGGTGGGCCTTTCTGCGTTTATATACTAGAGAGAGAATATAAAAAGCCAG
ATTATTAATCCGGCTTTTTTATTATTTTACTAGAGTTGACGGCTAGCTCAGTCCTAGGTACAGTGCTA
GCTACTAGAGAAAGAGGAGAAATACTAGATGACTATAATGATAAAAAAATCGGATTTTTGGCAATTC
CATCGGAGGAGTATAAAGGTATTCTAAGTCTTCGTTATCAAGTGTTTAAGCAAAGACTTGAGTGGGAC
TTAGTTGTAGAAAATAACCTTGAATCAGATGAGTATGATAACTCAAATGCAGAATATATTTATGCTTG
TGATGATACTGAAAATGTAAGTGGATGCTGGCGTTTATTACCTACAACAGGTGATTATATGCTGAAAA
GTGTTTTTCCTGAATTGCTTGGTCAACAGAGTGCTCCCAAAGATCCTAATATAGTCGAATTAAGTCGT
TTTGCTGTAGGTAAAAATAGCTCAAAGATAAATAACTCTGCTAGTGAAATTACAATGAAACTATTTGA
AGCTATATATAAACACGCTGTTAGTCAAGGTATTACAGAATATGTAACAGTAACATCAACAGCAATAG
AGCGATTTTTAAAGCGTATTAAAGTTCCTTGTCATCGTATTGGAGACAAAGAAATTCATGTATTAGGT
GATACTAAATCGGTTGTATTGTCTATGCCTATTAATGAACAGTTTAAAAAAGCAGTCTTAAATTAATA
ATACTAGAGTCACACTGGCTCACCTTCGGGTGGGCCTTTCTGCGTTTATATACTAGAGAGAGAATATA
AAAAGCCAGATTATTAATCCGGCTTTTTTATTATTTTACTAGAGTCCCTATCAGTGATAGAGATTGAC
ATCCCTATCAGTGATAGAGATACTGAGCACTACTAGAGAAAGAGGAGAAATACTAGATGCGTAAAGGA
GAAGAACTTTTCACTGGAGTTGTCCCAATTCTTGTTGAATTAGATGGTGATGTTAATGGGCACAAATT
TTCTGTCAGTGGAGAGGGTGAAGGTGATGCAACATACGGAAAACTTACCCTTAAATTTATTTGCACTA
CTGGAAAACTACCTGTTCCATGGCCAACACTTGTCACTACTTTCGGTTATGGTGTTCAATGCTTTGCG
AGATACCCAGATCATATGAAACAGCATGACTTTTTCAAGAGTGCCATGCCCGAAGGTTATGTACAGGA
AAGAACTATATTTTTCAAAGATGACGGGAACTACAAGACACGTGCTGAAGTCAAGTTTGAAGGTGATA
CCCTTGTTAATAGAATCGAGTTAAAAGGTATTGATTTTAAAGAAGATGGAAACATTCTTGGACACAAA
TTGGAATACAACTATAACTCACACAATGTATACATCATGGCAGACAAACAAAAGAATGGAATCAAAGT
TAACTTCAAAATTAGACACAACATTGAAGATGGAAGCGTTCAACTAGCAGACCATTATCAACAAAATA
CTCCAATTGGCGATGGCCCTGTCCTTTTACCAGACAACCATTACCTGTCCACACAATCTGCCCTTTCGA
AAGATCCCAACGAAAAGAGAGACCACATGGTCCTTCTTGAGTTTGTAACAGCTGCTGGGATTACACAT
GGCATGGATGAACTATACAAATAATAA

II. MICROSCOPE IMAGES OF CELL ELONGATION

Here we show the effects of $MinC$ expression on the cellular phenotype. Images shown were taken by fluorescent confocal microspy. For cells unable to synthesize their own lactone (p011, top), and cells with the full suite of genes (p111, bottom) we show the effect of H induced $MinC$ expression of cellular phenotype. Cells with internal $MinC$ imbalance can become hundreds of times longer than their wild-type counterparts and their biomass growth is reduced [4].

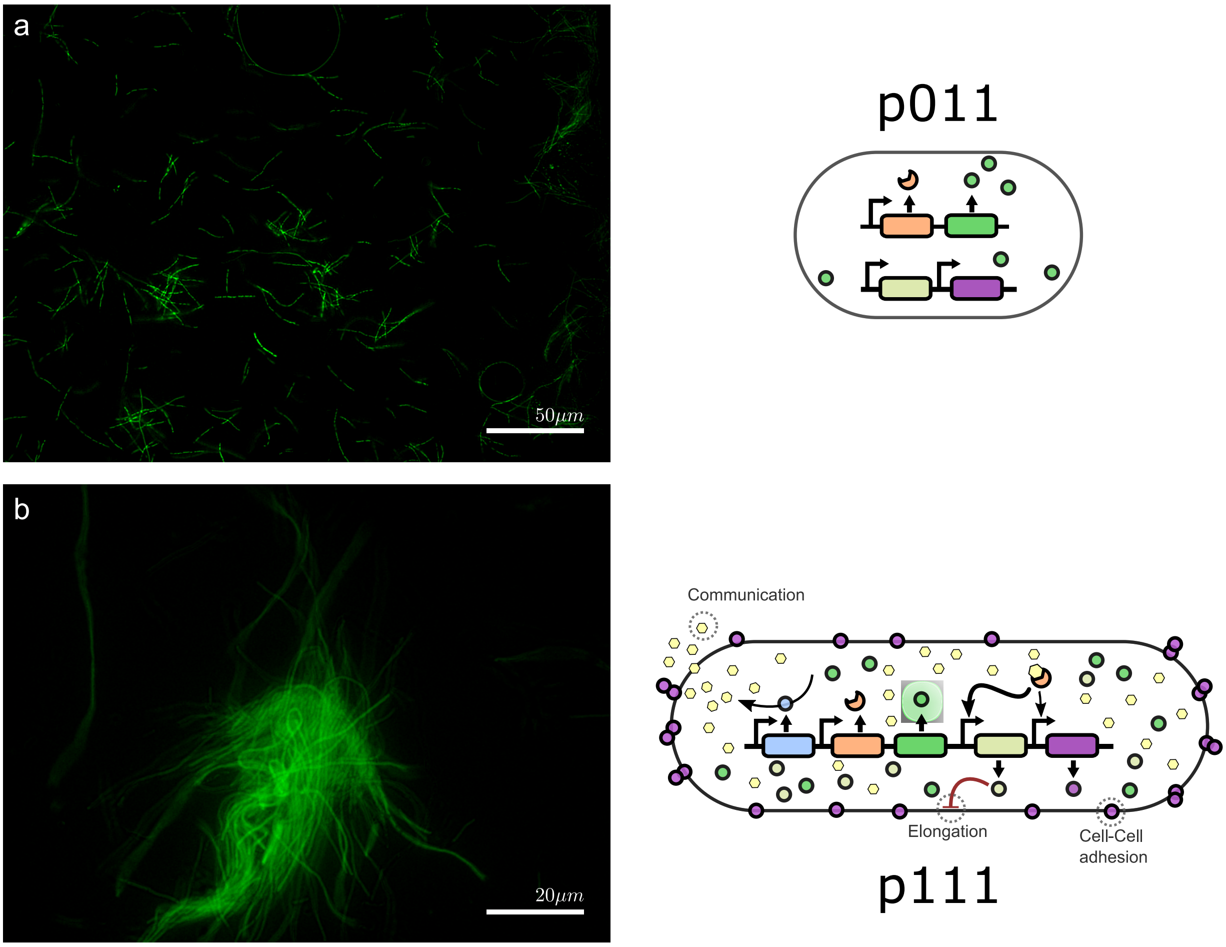

FIG. 3: **Effects of MinC differentiation**. On the left panels, p011 (top) and p111 (bottom) fluorescent microscopy images are shown. On the right, the genetic activation of MinC and lactone availavility is shown for each case.

III. LONG TERM EVOLUTION

Here we show a single p111 experiment after 14 days of uninterrupted growth at 22C. As the activated domain size increases it becomes unstable and new troughs of cell density are created close to the center of the branch. This colony shows multiple branchings produced at later stages of colony development.

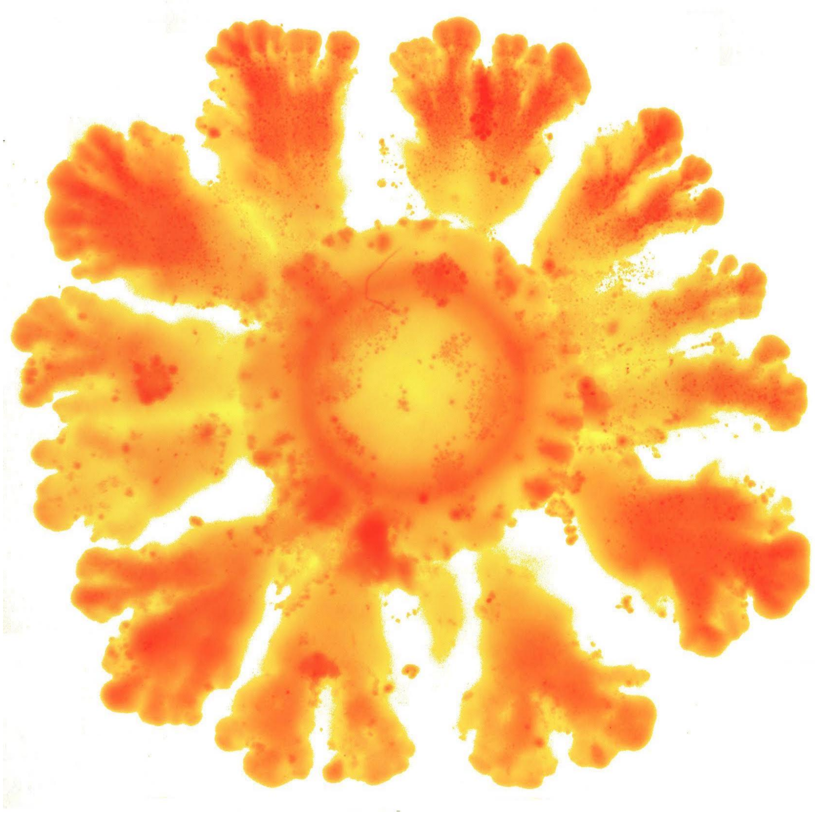

FIG. 4: **Long term evolution and branching in our system**. After 14 days of colony growth at 22C, established low-activity regions have not been blurred by homogenizing diffusion and new branching events have taken place.

IV. REFERENCES

[1] Shetty, R. P., Endy, D., & Knight, T. F. (2008). Engineering BioBrick vectors from BioBrick parts. **Journal of biological engineering**, 2(1), 1.

[2] Carbonell-Ballestero, M., Duran-Nebreda, S., Montañez, R., Solé, R., Macía, J., & Rodríguez-Caso, C. (2014). A bottom-up characterization of transfer functions for synthetic biology designs: lessons from enzymology. **Nucleic acids research**, 42(22), 14060-14069.

[3] Veiga, E., de Lorenzo, V., & Fernández, L. A. (2003). Autotransporters as scaffolds for novel bacterial adhesins: surface properties of Escherichia coli cells displaying Jun/Fos dimerization domains. **Journal of bacteriology**, 185(18), 5585-5590.

[4] Bhomkar, P., Materi, W., & Wishart, D. S. (2011). The bacterial nanorecorder: Engineering E. coli to function as a chemical recording device. PLoS One, 6(11), e27559.
